# The Autoimmunity Risk Variant LYP-W620 Cooperates with CSK in the Regulation of TCR Signaling

**DOI:** 10.1371/journal.pone.0054569

**Published:** 2013-01-24

**Authors:** María Luisa de la Puerta, Antonio G. Trinidad, María del Carmen Rodríguez, José María de Pereda, Mariano Sánchez Crespo, Yolanda Bayón, Andrés Alonso

**Affiliations:** 1 Instituto de Biología y Genética Molecular (IBGM), CSIC-Universidad de Valladolid, Valladolid, Spain; 2 Centro de Investigación del Cáncer, CSIC-Universidad de Salamanca, Salamanca, Spain; University Paris Sud, France

## Abstract

The protein tyrosine phosphatase LYP, a key regulator of TCR signaling, presents a single nucleotide polymorphism, C1858T, associated with several autoimmune diseases such as type I diabetes, rheumatoid arthritis, and lupus. This polymorphism changes an R by a W in the P1 Pro rich motif of LYP, which binds to CSK SH3 domain, another negative regulator of TCR signaling. Based on the analysis of the mouse homologue, Pep, it was proposed that LYP and CSK bind constitutively to inhibit LCK and subsequently TCR signaling. The detailed study of LYP/CSK interaction, here presented, showed that LYP/CSK interaction was inducible upon TCR stimulation, and involved LYP P1 and P2 motifs, and CSK SH3 and SH2 domains. Abrogating LYP/CSK interaction did not preclude the regulation of TCR signaling by these proteins.

## Introduction

LYP (lymphoid tyrosine phosphatase), encoded by the human gene *PTPN22*, is a classical protein tyrosine phosphatase (PTP) included in the group of PEST (Pro, Glu, Ser, and Thr) phosphatases [1], which also contains PTP-PEST and HSCF phosphatases. They share a highly similar N-terminal PTP domain and a Pro-rich motif (PRM) in the C-terminus called CTH (C-terminal homology domain). LYP and PTP-PEST present others PRMs, in addition to the CTH, In particular, LYP includes two other PRM: P1 motif (aa 615–620), and P2 motif (aa 690–700). Another characteristic to all the PEST phosphatases is the capacity to bind CSK, the kinase that regulates negatively Src family kinases (SFKs) [2].

LYP expression is restricted to hematopoietic cells. Studies on T lymphocytes have implicated this phosphatase in the regulation of TCR signaling pathways [3] where several proteins have been proposed to be LYP substrates, for example vav, the ζ chain [4], Cbl [5] and the kinases LCK, Fyn and Zap-70 [4], [6]. Among these proteins, the best characterized substrate of LYP is LCK, a SFK (Src family kinase) critical for T-cell development and activation. LYP dephosphorylates LCK Tyr394, the positive regulatory Tyr placed in its activation loop [4]. Another critical residue for LCK activity is the C-terminal Tyr505 that, when is phosphorylated by CSK, interacts intramolecularly with the SH2 domain and favors a closed and inactive conformation of LCK. It has been proposed that the concerted action of the tandem formed by Pep and CSK inactivates LCK [6], [7], [8].

The description in LYP of a single nucleotide polymorphism (SNP) [9], [10] associated to several autoimmune diseases such as type 1 diabetes, systemic lupus erytematosus and rheumatoid arthritis [11] indicates that this phosphatase plays a critical role in the regulation of the immune response. This SNP, C1858T, changes into a Trp the Arg620 present in the P1 PRM that binds to CSK SH3 domain [9], [12]. Based on data obtained in T lymphocytes, LYPW has been proposed to be a gain-of-function variant with increased phosphatase activity that reduces early T-cell signaling parameters such as Ca^2+^ mobilization and LCK phosphorylation [13]. Nevertheless, it is not fully clear how these changes in early signaling affect T cell physiology. A recent work has proposed that a reduced interaction with CSK leads to a lower tyrosine phosphorylation of LYP in a negative regulatory site, responsible for the increase in the activity of LYP [14]. Although the gain-of-function phenotype has received support from several studies, there is no agreement on this point; and recent reports have claimed that LYPW is a loss of function variant [15], [16]. Furthermore, knockout mice deficient in Pep phosphatase did not develop any autoimmune disease [17], despite augmented LCK activity in re-stimulated T-lymphocytes and an increase in the number of germinal centers.

Current knowledge about LYP/CSK binding is mainly based on the study of Csk interaction with Pep [6], [8], [12]. However, no detailed study has been yet reported on the association of LYP with CSK to determine the validity of this model in human cells, which is relevant to the pathogenesis of autoimmune diseases. Therefore, to determine how the R620W polymorphism modifies the function of this complex and contributes to the induction of autoimmunity, in this work we have analyzed LYP/CSK interaction and its relevance for TCR signaling.

## Materials and Methods

### Antibodies and Reagents

Tissue culture reagents were from Lonza (Verviers, Belgium). The anti-hemagglutinin (HA) mAb was from Covance (Berkely, CA, USA). The anti-LCK mouse Ab (3A5), anti-GST Ab, anti-myc Ab (9E10), anti-Erk2 Ab (C154), anti-Fyn Ab (6A406) and anti-CSK rabbit polyclonal Ab (C-20) were from Santa Cruz Biotechnology Inc. (Santa Cruz, CA, USA). The anti-CD3 (UCHT1), anti-CD28 (clone CD28.2), anti-Abl and anti-CSK mouse Ab were from BD Pharmingen (Franklin Lakes, NJ, USA). The anti-phosphotyrosine 4G10 mAb was from Millipore (Billerica, MA, USA).The anti-LYP goat polyclonal Ab was from R&D Systems, Inc. (Minneapolis, MN, USA). The anti-phospho-p38 Ab was from Cell Signaling Technology Inc., (Beverly, MA, USA).The anti-phospho-Erk Ab was from Promega (Fitchburg, WI, USA).

### Plasmids and Mutagenesis

Standard molecular biology techniques were used to generate the different constructions used in this study. Site-directed mutagenesis was done with the QuickChange Mutagenesis Kit (Agilent-Stratagene, CA, USA) following the manufacturer instructions. All constructions and mutations were verified by nucleotide sequencing.

### Cell Culture and Transfections

HEK293 were maintained at 37°C in Dulbecco’s modified Eagle’s medium supplemented with 10% FBS, 2 mM L-glutamine, 100 U/ml penicillin G, and 100 µg/ml streptomycin. Transient transfection of HEK293 cells was carried out using the calcium phosphate precipitation method [18]. JCam1.6, P116 and Jurkat T leukemia cells were kept at logarithmic growth in RPMI 1640 medium supplemented with 10% FBS, 2 mM L-glutamine, 1 mM sodium pyruvate, non essential aa, 100 U/ml penicillin G, and 100 µg/ml streptomycin. Transfection of Jurkat T cells was performed by electroporation as described previously [19]. PBLs were isolated from buffy coats of healthy donors obtained from the regional blood bank, with approval of its ethical committee, by centrifugation on Ficoll-Hypaque (GE Healthcare, Buckinghamshire, UK.) cushions. Monocytes/macrophages were eliminated by adherence to plastic for at least 1 h at 37°C.

### Immunoprecipitation, GST Pull-down, SDS PAGE and Immunoblotting

These procedures were done as reported before [19]. Briefly, cells were lysed in lysis buffer: 20 mM Tris/HCl pH = 7,4, 150 mM NaCl, 5 mM EDTA containing 1% NP-40, 1 mM Na_3_VO_4_, 10 µg/ml aprotinin and leupeptin, and 1 mM PMSF, pH 7.5, and clarified by centrifugation at 15,000 rpm for 10 min. The clarified lysates were preadsorbed on protein G-Sepharose (GE Healthcare, Buckinghamshire, UK.) and then incubated with Ab and protein G-Sepharose beads for 1 h. Immune complexes were washed three times in lysis buffer and suspended in SDS sample buffer. Proteins resolved by SDS-PAGE were transferred electrophoretically to nitrocellulose membranes, and immunoblotted with optimal dilutions of specific Abs, followed by the appropriate anti-IgG-HRP conjugate. Blots were developed by the enhanced chemiluminescence technique with Pierce ECL Western Blotting substrate (Thermo Scientific, Rockford IL, USA) according to the manufacturer’s instructions. Pull-down of GST fusion proteins was done with Glutathion sepharose beads (GE Healthcare, Buckinghamshire, UK.) incubated with the clarified lysates for 2 h. The complexes were then washed and processed as explained above for the IP. Blots were scanned with the GS-800 Densitometer (Bio-Rad Laboratories, CA, USA) and analyzed with the image analysis software Quantity One (Bio-Rad Laboratories, CA, USA). Data are reported as arbitrary units.

### Luciferase Assays

Transfection of Jurkat T cells and assays for LUC activity were performed as described previously [19], [20]. Briefly, 20×10^6^ Jurkat cells were transfected with 20 µg empty pEF vector or the indicated plasmids, along with 3 µg of NFAT/AP-1-luc (or other reporters) and 0.5 µg of a *Renilla* luciferase reporter for normalization. Cells were stimulated with anti-CD3 plus anti-CD28 Abs 24 h after transfection for the last 6 h. Cells were lysed then and processed to measure the LUC activity with the Dual Luciferase system (Promega, CA USA) according to the manufacturer’s instructions.

### Flow Cytometry and Immunohistochemistry

Jurkat cells were stimulatd with soluble anti-CD3 plus anti-CD28 Abs for 24 hours and were stained with Phycoerythrin (PE)-labeled anti-CD25 or PE-IgG2b isotype control (Immunostep, Salamanca, Spain). Data were acquired on a Gallios Flow Cytometer instrument (Beckman Coulter, Inc. CA, USA) and analysis was carried out with WinMDI software.

## Results

### LYP/CSK Binding in Human T Cells is Induced Upon T Cell Stimulation

**Figure 1:**
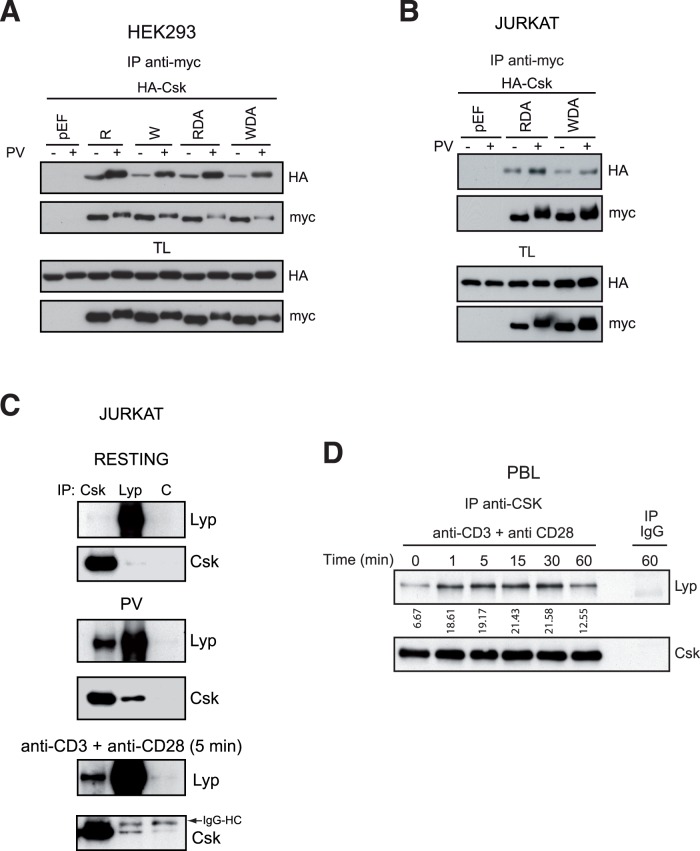
LYP binds to CSK in an inducible manner. *A*, Total lysates (TL) of HEK293 cells transiently transfected with LYP tagged with the myc epitope and HA-CSK, including the empty vector pEF as control, and treated or untreated with pervanadate (PV) for 5 min were subjected to immunoprecipitation (IP) and immunoblot (IB) with the indicated antibodies. *B*, Lysates from Jurkat cells transfected with plasmids that express myc-LYPR-DA or myc-LYPW-DA treated with PV for 5 minutes were subjected to IP with anti-myc Ab and then blotted with anti-HA (upper panel) to detect CSK. Similar expression of the proteins in the assay was detected by IB in the TL. *C*, Lysates of Jurkat cells untreated (resting cells), treated with PV or stimulated with anti-CD3 and anti-CD28 Abs for 5 min were subjected to IP with anti-CSK Ab, anti-LYP Ab, or an irrelevant Ab used as control, and inmunoblotted against endogenous LYP and CSK. *D*, T lymphocytes obtained from peripheral blood of healthy donors were incubated for the indicated times at 37°C with medium alone or in the presence of anti-CD3 and anti-CD28 Ab. Lysates from these cells were immunoprecipitated with anti-CSK or an irrelevant Ab (IgG) to show specificity, and the presence of LYP and CSK in the precipitates was detected with specific Abs by IB. LYP blot was measured by densitometry and the values obtained, shown under the blot, are expressed in arbitrary units.

To verify the validity of the Pep/Csk cooperative model [6] for LYP/CSK interaction, we first tested in HEK293 cells the association of CSK with Arg620 and Trp620 LYP variants, in an active or inactive state (D195A substrate trapping mutant, referred throughout this paper as DA). In contrast with previous data for Pep [9], [21], we found that LYPW did bind CSK (Figure 1A), in agreement with data obtained for LYP [10], [14]. Thereafter, we tested whether cell activation could affect this interaction. Treatment of cells with pervanadate (PV), a potent PTP inhibitor, increased the binding of CSK and LYP either active or inactive, but the interaction of CSK with LYPW was always lower than with LYPR (Figure 1A). To confirm these results in a cell line more relevant to LYP function, we expressed LYP variants along with CSK in Jurkat cells, a well-known model for the study of early TCR signaling. In these cells, LYPW also interacted with CSK (Figure 1B) and, as before, this interaction was increased after PV treatment. IP of either LYP or CSK in Jurkat cells resulted in a very low co-precipitation of the other protein in resting cells (Figure 1C, upper panel); however, this association augmented after PV treatment (Figure 1C, middle panel) or TCR stimulation (Figure 1C, lower panel). Additionally, we verified that LYP/CSK interaction between endogenous proteins was increased upon CD3 and CD28 co-stimulation in PBLs (Figure 1D). The efficiency of stimulation in these cells was checked by Western blot with anti-PY Ab (Figure S1). Stimulation upon CD3 cross-linking alone also increased LYP/CSK interaction in a similar way to CD3 and CD28 co-stimulation (Figure S2). From these data, we concluded that, while Pep/CSK interaction is constitutive, the interaction between LYP and CSK could be induced by cellular activation. It is also worthy to mention the existence of a shift in the band that corresponds to LYP in cells treated with PV (Figure 1A and B) that can be most likely explained by LYP phosphorylation.

### P1 and P2 LYP Motifs Bind to CSK

**Figure 2:**
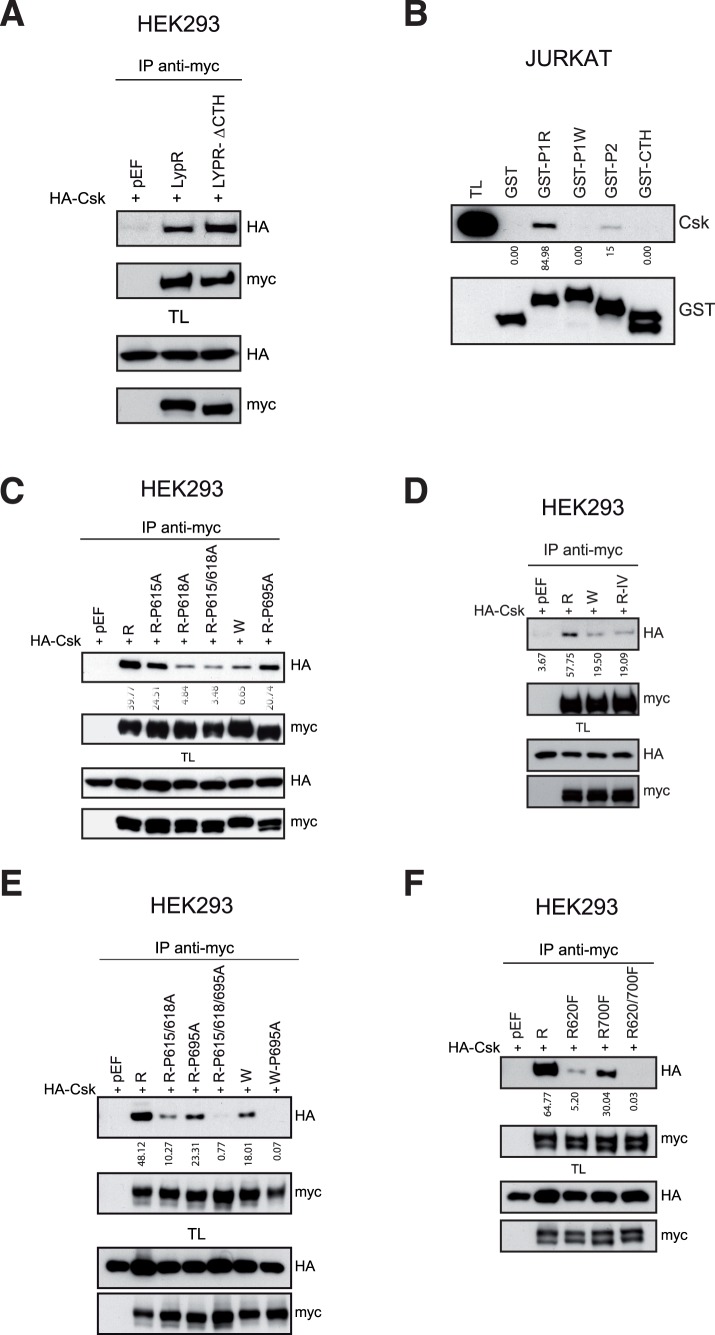
P1 and P2 LYP motifs bind to CSK. *A*, Lysates of HEK293 cells transfected with LYP and a deletion mutant of LYP tagged with the myc epitope that lacks the CTH PRM, tagged with the myc epitope, along with HA-CSK plasmids were immunoprecipitated and immunoblotted with the indicated Abs. Expression of these proteins was verified by IB in TL. *B*, Lysates from 3×10^8^ Jurkat cells were divided equally into five tubes and were subjected to pull-down assays with the indicated PRMs fused to GST. The presence of CSK in the precipitates was detected by IB with CSK Ab and GST-peptides were detected using a GST Ab. TL shows the presence of CSK in the lysate. *C*, Lysates of HEK293 cells transfected with different LYP mutants in the P1 and P2 motifs, tagged with the myc epitope, along with HA-CSK were subjected to IP and IB as indicated. *D*, Interaction of HA-CSK with a myc-LYP mutant in the C-terminus of the P1 motif, IV, (Ile626Ala,Val627 Ala) was verified by IB after LYP IP in transiently transfected HEK293 cells. *E*, Lysates of HEK293 cells transfected with HA-CSK and different mutants of LYP in the P1, the P2, or in both motifs, tagged with the myc epitope, were immunoprecipitated and immunoblotted with the indicated Abs. *F*, Arg to Phe LYP mutants in P1 and P2 PRM tagged with myc were expressed in HEK293 cells and interaction with HA-CSK was detected by immunoblot after LYP immunoprecipitation. CSK blots in panels B, C, D, E and F were scanned and the values obtained were expressed as arbitrary units under the blot.

The previous data suggested that either Arg620 is less critical than expected for CSK binding or CSK binds LYP through additional PRMs. In fact, LYP, as Pep, contains two additional motifs, the P2 motif, which shows a high similarity with the P1 motif, and the CTH motif. To discard the implication of the CTH motif we tested the interaction of CSK with a mutant of LYP lacking this motif (LYP-ΔCTH) by IP. In these assays, CSK was precipitated by LYP-ΔCTH in a similar way to LYP (Figure 2A). Furthermore, to determine which PRM binds CSK, we fused them with GST and produced the recombinant proteins in bacteria. Pull-down assays of Jurkat cell lysates with these fusion proteins showed that while P1W and CTH motifs did not bind, P1R and P2 motifs did bind to CSK (Figure 2B), the later with lower affinity.

To define the contribution of different residues in P1 and P2 LYP motifs to CSK binding, we mutated key residues of these motifs: Pro615, Pro618, R620W in P1 motif, and Pro695 and Arg700 in P2 motif. We tested the association of CSK with these mutants by co-IP assays in HEK293 cells transiently transfected. Unlike the data reported on Pep [21], none of the point mutants blocked completely LYP/CSK association. The single mutants that showed a lower binding were P618A and R620W polymorphism (Figure 2C). As other studies indicated that residues in the C-terminus of the P1 motif of Pep [8], equivalent to Ile626 and Val627 in LYP, contributed to Pep/CSK association, we replaced these aa by Ala (R-IV) and tested whether their mutation could abolish CSK/LYP binding. Again, whereas mutation of these residues in Pep blocked its association to CSK [8], I626A and V627A substitutions reduced, but did not abolish, LYP/CSK binding (Figure 2D). Therefore, based on the evidence collected so far, we reasoned that CSK also bound to LYP through the P2 motif; and that to abolish this association, both P1 and P2 motifs should be mutated. This hypothesis was tested by co-IP assays in which mutation of both motifs did abolish the association of CSK and LYP proteins in any of the combinations used, either P615A/P618A/P695 or W-P695A (Figure 2E). Furthermore, to confirm these data using different mutations, we replaced Arg620 and Arg700 by Phe, based on the information provided by the ADAN database [22], which predicts Phe as one of the aa with the lowest affinity in that position of P1 peptide. As before, only the double mutant completely abrogated LYP/CSK binding (Figure 2F). Altogether, these data indicated that CSK binds to two different motifs in LYP, P1 and P2, of which P1 showed a higher affinity. Accordingly, the change of Arg620 by a Trp reduced but did not abolish CSK binding.

### CSK SH2 and SH3 Domains are Involved in Binding to LYP

**Figure 3:**
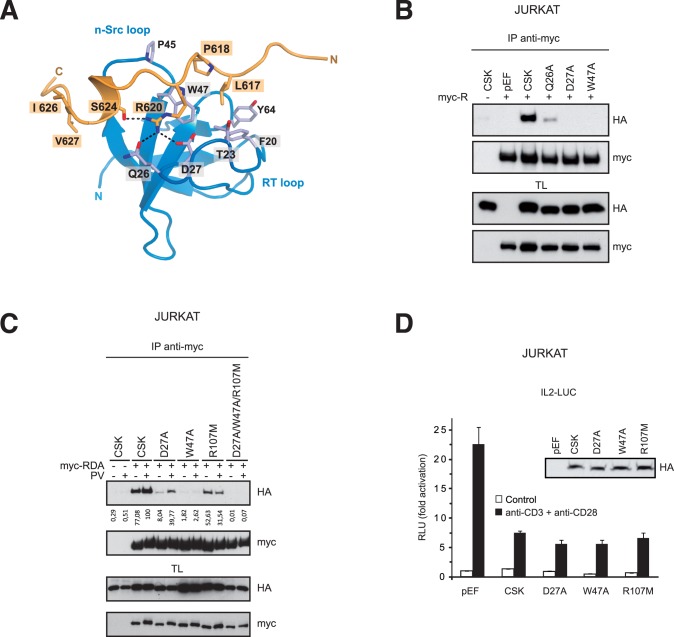
CSK SH3 and SH2 domains are involved in the association with LYP. *A* **,** Schematic representation of the NMR structure of the Pro rich motif P1 of Pep (orange) bound to the SH3 domain of CSK (blue) (PDB code 1JEG). P1 resid ues are numbered according to the LYP sequence (613IPPPLPVRTPESFIVVEE630). Arg620 is involved in intermolecular polar contacts with Asp27 and Gln26 of CSK; hydrogen bonds are shown by dashed lines. In addition, Arg620 could establish an intramolecular hydrogen bond with Ser624. *B*, Jurkat cells were electroporated with HA-CSK wild type and several mutants of CSK SH3 domain along myc-LYPR as indicated. Interaction was detected by IB after LYP IP. *C*, Several HA-CSK mutants in the SH3 and SH2 domains were tested for interaction with myc-LYP-RDA. Jurkat cells were left untreated or treated with PV and LYP was immunoprecipitated from cell lysates. CSK interaction was detected by IB with specific anti-HA antibody. *D*, Activation of a luciferase reporter gene driven by the IL-2 minimal promoter in Jurkat cells cotransfected with different CSK plasmids, as indicated. The insert shows the IB of the CSK proteins expressed.

The LYP residues that contribute to CSK binding have been studied largely. However, less attention has been paid to the CSK aa critical for this interaction. To address this issue, we generated D27A and W47A CSK mutants, which interact with Arg620 and Pro618 in LYP, respectively [23]. In addition, a careful examination of the NMR models of Ghose et al. suggested that Gln26 could form a hydrogen bond with Arg620 in LYP (Figure 3A), which prompted us to mutate Gln26 to Ala and test its binding to LYP. We observed that while D27A and W47A mutants blocked the association with LYP, the Q26A mutant seems less critical for this association (Figure 3B). Given that LYP/CSK interaction was increased by PV treatment (Figure 1A), we asked whether the SH2 domain of CSK was involved in the interaction with LYP. To prove this, we mutated CSK Arg107 to Met, because this residue, conserved in SH2 domains, is critical for binding to phospho-Y in protein ligands [24]. The R107M mutation decreased the association of CSK with LYP in cells treated with PV, but also in resting cells (Figure 3D). Whereas W47A mutation abolished the interaction with LYP, as did the triple mutant D27A/W47A/R107M; binding of CSK-D27A to LYP was increased after PV treatment, indicating that D27A mutant still binds LYP and that PV treatment increased this binding, (Figure 3D). Collectively, these data showed that the SH2 domain of CSK participates in the association with LYP, although the primary interaction is established with the CSK SH3 domain, in which is critical the Trp47. In addition, we tested the CSK mutants generated to study the interaction of this kinase with LYP in functional assays in Jurkat cells (Figure 3D). These mutants did not affect the negative role played by CSK in the regulation of TCR signaling, suggesting that CSK regulation of TCR signaling is not dependent on LYP binding.

### Relevance of LYP/CSK Interaction for TCR Signaling

**Figure 4:**
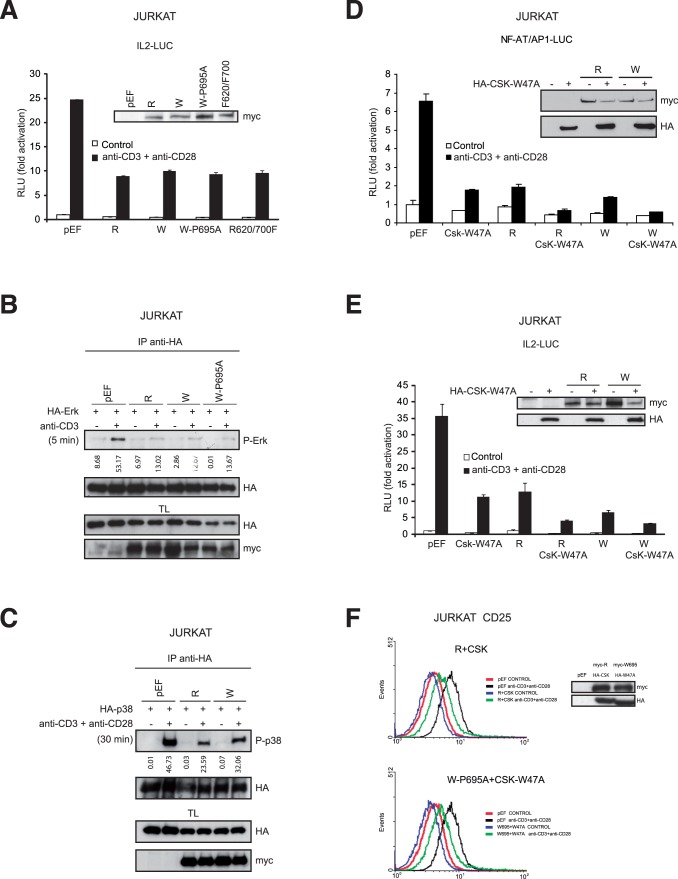
LYP/CSK interaction is not required to regulate TCR signaling. *A*, Activation of a luciferase reporter gene driven by the IL-2 minimal promoter in Jurkat cells co-transfected with different myc-LYP plasmids, as indicated. The insert shows the expression of the LYP proteins as detected by IB. *B*, Erk activation was assayed in Jurkat cells transfected with different versions of LYP, as indicated, and stimulated with anti-CD3 Ab for 5 min. Erk was immunoprecipitated from lysates of these cells and its phosphorylation was detected by IB. Expression was verified in total lysates (TL) by IB. Phospho-ERK (P-Erk) blot was measured by densitometry and the data were expressed as arbitrary units under the blot.*C*, As in B, p38 activity was evaluated in Jurkat T cells stimulated with anti-CD3 and anti-CD28 Ab for 30 min by IP of HA-p38 and IB with a specific antibody for dually phosphorylated p38. Phospho-p38 (P-p38) blot was measured by densitometry scanning and the data were expressed as arbitrary units under the blot. *D*, Activation of a luciferase reporter gene driven by the NF-AT/AP1 site of IL-2 promoter in Jurkat cells co-transfected with LYPR, LYPTW, and CSK-W47A plasmids, as indicated. Expression of LYP and CSK proteins as detected by IB is shown in the insert. *E*, Activation of a luciferase reporter gene driven by the IL-2 minimal promoter in Jurkat cells transfected with LYPR, LYPW, and CSK-W47A plasmids. The insert shows the IB of LYP and CSK proteins. R, LYPR; W, LYPW. *F,* Expression of CD25 in Jurkat cells transfected with the plasmids indicated was measured by flow cytometry upon stimulation with anti-CD3 plus anti-CD28 antibodies for 24 hours. Expression of LYP and CSK proteins as detected by IB is shown in the insert.

A consequence of the LYP C1858T polymorphism is a reduction in CSK binding that can be the cause of the alterations produced by this variant in the immune system. To determine how this polymorphism affects TCR signaling, and to evaluate the importance of LYP/CSK interaction in these pathways we used several CSK and LYP mutants that bind to each other in different degrees. First, we carried out luciferase assays in Jurkat cells to compare the activity of LYPR and LYPW in different promoters relevant for T cells, such as NF-AT/AP1 sites of the IL-2 promoter, Gal4-ELK or NF-κB (Figure S3). We observed in these assays that LYPW had a similar activity than LYPR. Next, we observed that LYPW-P695A and LYP-F620A/F700A, which are mutated in both P1 and P2 motifs and do not interact with CSK, inhibited the activation of the IL-2 minimal promoter as did LYPR and LYPW (Figure 4A). We also determined the activation of Erk in the presence of LYPR, LYPW or LYPW-P695A (Figure 4B) and the activation of p38 in the presence of LYPR or LYPW (Figure 4C), obtaining a similar inhibition, in agreement with the data obtained in the luciferase assays. Although the inhibitory role of LYPW in these assays could have been explained by the binding to endogenous CSK through the P2 motif, LYPW-P695A and LYP-F620A/F700A do not bind to CSK at all, precluding any effect of the interaction of LYP with endogenous CSK in these assays. In addition, to address the cooperation of LYP and CSK in TCR signaling, we expressed CSK along with LYPR and LYPW to measure the induction of a promoter with NF-AT/AP1 sites of IL-2 (data not shown). In both cases CSK cooperated with LYPW as well as with LYPR, and in both cases co-expression of CSK and LYP produced a greater inhibition than any of the proteins alone. Likewise, when CSK-W47A, which showed no binding to LYP, was co-expressed with LYPR and LYPW there was an increase of the inhibition of the NF-AT/AP1 and IL-2 minimal promoter (Figure 4D and E). We also tested whether LYP and CSK versions that do not interact to one another could reduce the expression of CD25 in Jurkat cells. This experiment showed that a combination of plasmids like LYPW-P695A and CSK-W47A, are still able to reduce the induction of this activation marker (Figure 4F). Collectively, we conclude from these data that the cooperation of LYP and CSK proteins to regulate TCR signaling does not require a direct interaction between them.

### LYP is Phosphorylated in Tyrosine

**Figure 5:**
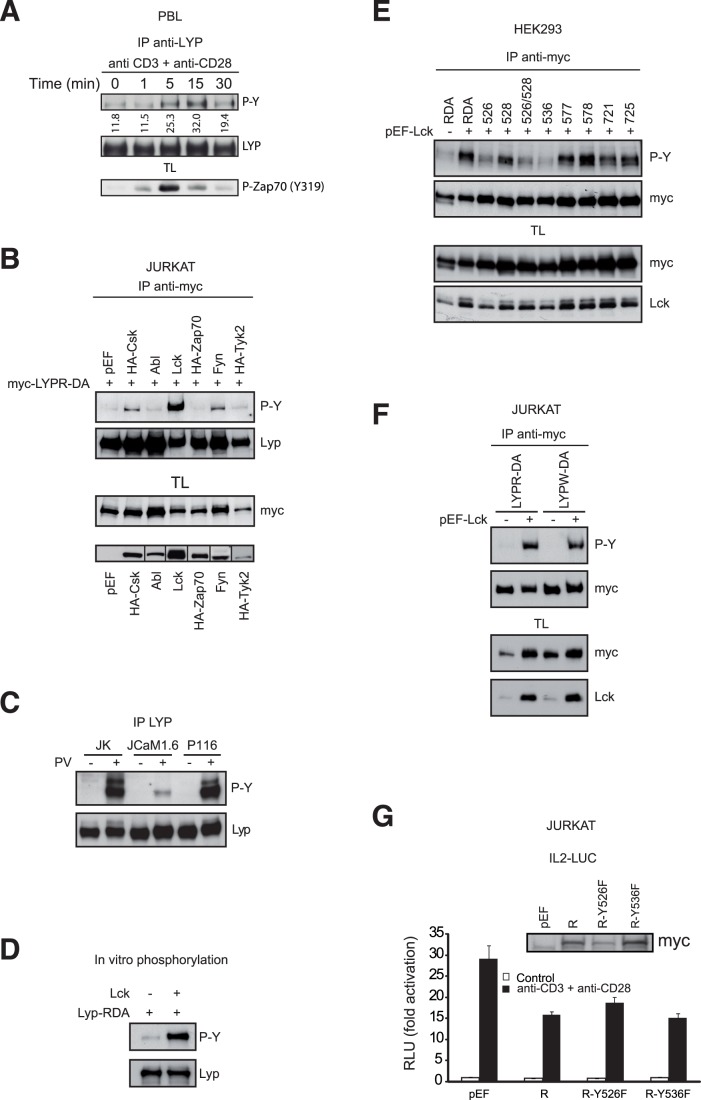
LYP is phosphorylated in tyrosine. *A*, Lysates of PBLs stimulated with Abs against CD3 and CD28 molecules were subjected to IP with anti-LYPAb and immunoblotted with anti-phospho-Y Ab 4G10 to detect LYP Y-phosphorylation. After stripping, the membrane was probed with anti-LYP Ab to verify equal loading of LYP. Zap70 phosphorylation with anti-Phospho-Y319 was tested to check activation of the cells. The blot showing LYP Tyr phosphorylation (P-Y) was measured by densitometry and the data were expressed as arbitrary units under the blot. *B*, Lysates of Jurkat cells transfected with myc-LYPR-DA and the indicated kinases were subjected to IP to detect Lyp Y-phosphorylation as in A. Expression of the kinases transfected was detected by Western blot with anti-HA antibody, where HA was present, or with specific antibodies for the untagged kinases. *C*, LYP Y-phoshophorylation was studied in different Jurkat derived cell lines deficient in LCK (JCaM1.6) and Zap70 (P116) for comparison with Jurkat parental cells. Phosphorylation of endogenous LYP after IP was detected as aforementioned. *D*, In vitro phosphorylation of myc-LYP-RDA by recombinant LCK, LYP was immunoprecipitated from HEK293 transfected cells and active recombinant LCK was added to the beads along with ATP and the kinase buffer. The reaction was incubated at 30°C for 30 min. and LYP phosphorylation was detected as before. *E*, HEK293 cells were transfected with several myc-LYPR-DA Tyr to Phe mutants along with LCK. Phosphorylation of LYP was detected by IB with 4G10 Ab after IP of LYP. *F*, Lysates of Jurkat cells transfected with myc-LYPR-DA or myc-LYPW-DA along with LCK were subjected to IP and phosphorylation was detected as before. *G,* Activation of a luciferase reporter gene driven by the IL-2 minimal promoter in Jurkat cells cotransfected with different LYP plasmids, as indicated. The insert shows the expression of LYP proteins by IB.

As PV treatment produced a shift in LYP SDS-PAGE mobility (Figure 1A), we speculated that this shift could be due to Tyr phosphorylation. To address this issue, a PBLs were stimulated through CD3 and CD28 receptors, and LYP was shown to be phosphorylated in tyrosine by Western blot (Figure 5A). Next, to determine the kinase(s) responsible for this phosphorylation, we co-expressed in Jurkat cells LYPR-DA with several kinases relevant for T cells (Figure 5B). Co-expression of LCK, Fyn, and CSK lead to LYP Tyr phosphorylation, being the highest phosphorylation produced by LCK. To confirm that LCK was the main kinase involved in LYP phosphorylation, we used Jurkat derived cell lines deficient in LCK, JCam1.6, and in Zap70, P116 (Figure 5C). PV treatment of Jurkat and P116 cells lead to similar levels of LYP Tyr phosphorylation, while in JCam1.6 cells there was a residual phosphorylation that can be explained by the presence of FYN or CSK in these cells. We also detected *in vitro* LYP phosphorylation by LCK (Figure 5D), further supporting LCK as a key kinase in LYP tyrosine phosphorylation. Our analysis on LYP phosphorylation was followed by the identification of the tyrosines phosphorylated. To this end, we transfected several Tyr to Phe mutants, in a LYPR-DA inactive version, chosen based on the phosphorylation sites predicted by Netphos [25] or Scansite [26], and on the degree of evolutionary conservation. Co-transfection of LCK with the LYP Tyr to Phe mutants showed that the main sites phosphorylated by LCK were Tyr526 and Tyr536 (Figure 5E). We also tested whether there was any difference in the phosphorylation of LYPR and LYPW in Jurkat cells by LCK, which in fact was similar (Figure 5F). Then, we evaluated whether LYP phosphorylation on these residues, Tyr526 and Tyr536, was involved in the regulation of TCR signaling. Expression of LYP Y526F and Y536F mutants showed no effect with respect of LYPR on the activation of the IL-2 promoter in luciferase assays (Figure 5G), in disagreement with data published recently [14]. We also tested whether these mutants affected the interaction of LYP with CSK, but our results showed that they are not involved in this interaction (Figure S4),These results indicate that TCR stimulation leads to Y-phosphorylation of LYP, and that phosphorylation of Tyr526 and Tyr536 does not affect LYP function during TCR signaling.

## Discussion

The C1858T polymorphism of LYP plays a critical role in the pathogenesis of several autoimmune diseases. This polymorphism changes to a Trp the Arg620 that is essential for LYP binding to CSK, suggesting that a change in the association between these proteins will contribute to the generation of autoimmunity. Here, we analyzed the association between LYP and CSK, and its relevance to TCR signaling. Our work suggests that LYP/CSK interaction is more complex than expected from the data reported on Pep/Csk binding. Whereas Pep/Csk interaction is constitutive [6], LYP binding to CSK is dynamic and is increased by cellular activation, involving CSK SH3 and SH2 domains along with LYP P1 and P2 motifs. Extensive mutation of critical residues in the P1 motif: Pro615, Pro618, Arg620, Ser624, Ile626, Val626 did not abolished CSK binding to LYP, differing from the data reported on Pep [8]. Our data also show that CSK does bind to LYPW. Although this observation is not entirely novel, it is novel the demonstration that this binding is mediated by the P2 motif of LYP. Again, this is another controversial fact regarding LYP. The study of Pep/Csk interaction showed initially that a weak interaction exists on a yeast two-hybrid assay when the P1 motif is deleted [12]. Another work reported that mutations in the P1 motif of Pep abrogated Csk binding [8]. Later on, it was found that Pep binds to Csk after deletion of the P1motif in stable cell lines generated with different constructs of Pep [6]. Other reports have not detected LYPW interaction with CSK [27], [28]. However, in agreement with our data, other studies have reported that LYPW interacts with CSK [10], [14]. Particularly, the work of Fiorillo et al. showed this interaction in vitro between recombinant proteins. These last studies, as well as the data here presented, show that the affinity of LYPW by CSK is lower than that of LYPR, in parallel to the affinity of the P1 and P2 motifs, and the low affinity of the P2 motif may be the cause of the difficulties found to detect this interaction.

A complementary view of the association between LYP and CSK was obtained with the study of the CSK residues critical for this association. Thus, Trp47, in the CSK SH3 domain, is the key CSK residue for this interaction, as the sole mutation W47A abolished LYP-CSK interaction. Asp27 seems less critical for this interaction because a CSK D27A mutant still binds LYP and, more important, binding to LYP was increased after PV treatment. Moreover, we showed the involvement of an additional CSK residue, Gln26, which shows polar contacts with Arg620 in LYP (Figure 3A). In addition to the SH3 domain, our data support the implication of CSK SH2 domain in LYP/CSK association, because the SH2 CSK mutant, R107M, reduces this interaction. In particular, the SH2 domain appears to be responsible for the increase observed in the association between these proteins upon TCR engagement. The fact that the SH2 CSK mutant, R107M, also showed a lower association to LYP in resting cells could be due to the existence of basal LYP phosphorylation or to an alternative mechanism of interaction, currently unknown, that might involved the interaction with a third protein. LYP is not the first PEST PTP in which tyrosine phosphorylation is involved in binding to CSK, as it has been already shown that PTP-HSCF binds to CSK SH2 domain [29].

The CSK mutants used in this study clearly affect the association of this kinase with LYP, as we have shown throughout this paper; however they do not differ much from CSK wild type in the regulation of TCR signaling (Figure 3D), in contrast with previous works, which have reported that deletion of these domains in CSK abrogates TCR signaling [7], [30], [31]. It can be argued that point mutation, in contrast to domain deletion, is expected to produce a lower effect in the overall structure of the protein while affecting ligand binding. In fact, another study pointed that CSK SH3 and SH2 domains are critical for CSK to show full kinase activity [32]; and the recent structural data on the Src/CSK complex shows that SH3 and SH2 CSK domains are not involved in Src recognition by CSK [33]. The role of CSK SH2 domain in the negative regulation of TCR signaling is controversial because despite being implicated in CSK recruitment to the plasma membrane through the association with PAG/Cbp, as judged from a flurry of biochemical data; however, knockout mice for PAG/Cbp showed no alteration in T cell activation and development [34], [35]. The same was true for LIME, another membrane adaptor related to PAG that interacts with CSK by a similar mechanism [36].

The regulatory function of LYP on TCR signaling is well documented. However, the consequences of the R620W SNP for T cell function remain controversial. Initially, it was proposed that LYPW was a gain-of-function variant of this PTP [13]. The gain of function of LYPW has been mainly ascribed to the initial steps of antigen signaling in T cells, being less clear at later steps, for example IL-2 production [13], [37], [38], [39] or T cell proliferation [37]. On the contrary, other reports suggested that LYPW is a loss of function variant [15], [16]. In the present study, we have found that LYPW behaves similarly to LYPR in the context of TCR signaling. Therefore, our data support a third possibility, i.e., LYPW is neither a gain- nor a loss-of-function in the context of TCR signaling. According to our results, mutations that reduced or abolished this interaction do not affect to the capacity of these proteins to regulate TCR signaling. Thus, a combination of mutants like CSK-W47A and LYPW still cooperate to further reduce TCR signaling indicating that cooperation of LYP and CSK on TCR signaling is not based on a direct physical interaction. In this sense, it is worthy to mention here that removal of the CSK binding motif in PTP-PEST, another PEST phosphatase, had no consequence for PTP-PEST regulatory role in B cells [40]. A recent work has shown that overexpression of CSK SH3 domain reduces TCR signaling, effect that the authors explained by its inhibition of the interaction between endogenous LYP and CSK. These data show that LYP inhibition of TCR signaling does not require CSK binding, in agreement with our data.

A change in the mobility of LYP in SDS-PAGE after PV treatment prompted us to study LYP phosporylation. In this respect, we have shown that LYP is phosphorylated on Tyr upon TCR stimulation, being LCK the kinase mainly responsible for LYP phosphorylation in T cells. Our data on LYP phosphorylation agrees with a recent report [14], although there are discrepancies, for example in the kinetics of LYP phosphorylation, which seems delayed in our assays, probably due to the different cell lines used in each case. The major sites phosphorylated by LCK appeared to be Tyr526 and Tyr536. However, it remains elusive the role of Tyr phosphorylation for LYP function in TCR signaling, as mutation of several Tyr to Phe, including Tyr526 and Tyr536, did not alter the negative regulatory role of LYP in TCR signaling.

In summary, the data collected in this report reveals that LYP/CSK interaction is dynamic and is not based solely on a direct binding between a PRM and an SH3 domain, being additional mechanisms involved in this interaction. Furthermore, the interaction of CSK and LYP in resting cells is increased upon TCR engagement by a mechanism that implicates the SH2 domain of CSK and probably LYP Tyr phosphorylation by LCK. Although the critical role played by LYP in TCR signaling is well documented, it is far from being clear the function of the LYP/CSK complex as well as the consequences of the R620W polymorphism for T cell physiology. In this regard, it will be essential clarifying the physiological role played by LYP in the immune system to determine how LYP function is altered by this polymorphism.

## Supporting Information

Figure S1 **Activation of PBLs tested by Western blot.** Lysates corresponding to the experiment shown in Figure 1D were immunoblotted with anti-PY Ab to show stimulation of the cells used in this experiment.
(EPS)

Figure S2 **LYP/CSK interaction by TCR stimulation.** T lymphocytes obtained from peripheral blood of healthy donors were incubated for 15 minutes with medium alone as control (C), in the presence of anti-CD3, or with anti-CD3 and anti-CD28 Abs. Lysates from these cells were immunoprecipitated with anti-CSK Ab, and the presence of LYP and CSK in the precipitates was detected with specific Abs by IB. LYP blot was measured by densitometry and the data were expressed as arbitrary units under the blot.
(EPS)

Figure S3 **Activation of different luciferase promoters in the presence of LYPR and LYPW.** Activation of a luciferase reporter gene driven by NF-κB (A), Gal-4-ELK (B) or the NF-AT/AP1 sites of the IL-2 promoter (C) in Jurkat cells co-transfected with different myc-LYPR (R) or myc-LYPW (W) plasmids, as indicated. The insert in each panel shows the expression of the LYP proteins as detected by IB.
(EPS)

Figure S4 **Tyrosines 526 and 536 are not involved in CSK interaction.** Interaction of HA-CSK with myc-LYP mutants, Y526F and Y536F was tested by IB after LYP IP in transiently transfected HEK293 cells treated with PV.
(EPS)
